# Lectures on Neurology and Neuriatry, Psychology and Psychiatry

**Published:** 1904-05

**Authors:** 


LECTURES ON NEUROLOGY AND NEURIATRY, PSYCHOLOGY AND PSYCHIATRY. After the Methods of the class-room, to the Author's Students, and Designed also for General Practitioners of Medicine and Surgery. By C. H. HUGHES, M. D., President of Faculty and Professor of Neurology, Psychiatry and Electrotherapy, Barnes Medical College, Saint Louis. Edited by Prof. Marc Ray Hughes, M. D. Octavo, pages 417. Illustrated. Saint Louis: Hughes & Co. 1903. (Price, $3.00.)

The author has not aimed to present the various diseases of the nervous system, hence we do not find here a description of the well recognised types of cerebral, spinal, peripheral and functional diseases. He has chosen rather to present the method of examination of neurological cases. The principles which govern electro-diagnosis and therapeutics are given careful attention. The use of lumbar puncture in diagnosis is also accorded adequate consideration.

The book can hardly be called a guide for the practitioner, for the treatment of diseases forms but a small part of the author's plan. It is rather as a representation of the individual views of the author on many of the problems of neurology that this book is to be commended. J. W. P.

